# Supplementary material for: Curated and harmonised transcriptomics datasets of interstitial lung diseases
Source: Data Brief. 2025 Oct 14;63:112139. doi: 10.1016/j.dib.2025.112139 (PMC12581653; doi:10.1016/j.dib.2025.112139)
Supplement: Supplementary file 1 [file mmc1.zip › Supplementary_material/DNA-microarray/GSE53845/GSE53845_eUTOPIA_Agilent_QC_Report_2023-12-15.tar.gz!/Users/OWNER/AppData/Local/Temp/RtmpAFKb7F/2023-12-15/pca.pdf]

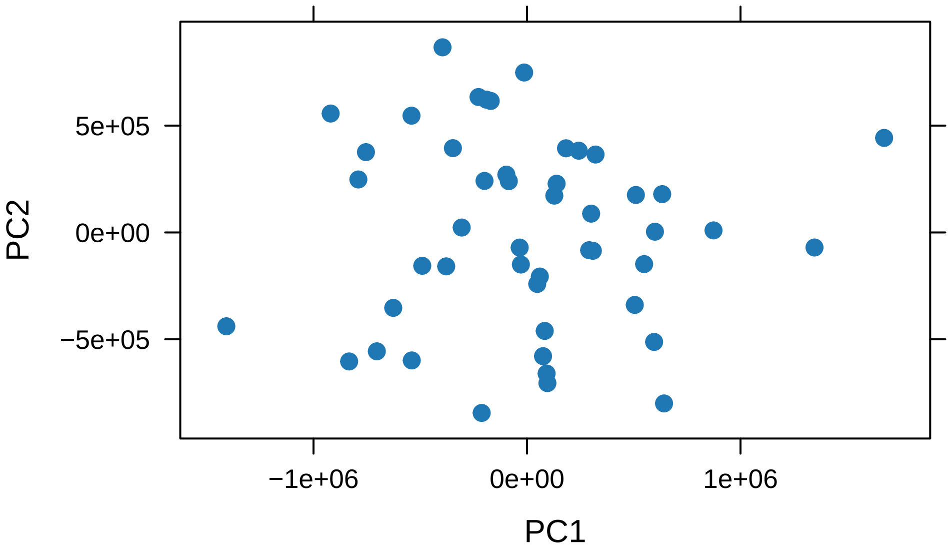

PC2
5e+05
0e+00
−5e+05
−1e+06
0e+00
1e+06
PC1
